# Tuberculosis infection control in health care facilities in Enugu State, Nigeria: a cross-sectional facility-based study

**DOI:** 10.11604/pamj.2022.41.181.30114

**Published:** 2022-03-07

**Authors:** Lawreta Ijeoma Abugu, Tochi Emmanuel Iwuagwu, Eunice Nguungwan Seer-Uke, Wamanyi Yohanna, Ifunanya Rosemary Obi, Dorothy Nwakaego Eze, Samuel Ifeanyichukwu Onuorah

**Affiliations:** 1 Department of Human Kinetics and Health Education, University of Nigeria Nsukka, Nsukka, Nigeria,; 2 Benue State University, Makurdi, Nigeria,; 3 Adamawa State Primary Healthcare Agency, Adamawa, Nigeria,; 4 Federal College of Education (Technical) Umunze, Anambra State, Nigeria,; 5 Nsukka Health Centre, Nsukka, Enugu State, Nigeria

**Keywords:** Opportunistic infection, infection control, implementation, collaborative TB/HIV activities

## Abstract

**Introduction:**

overtime, tuberculosis (TB) has remained the most common opportunistic infection among people living with HIV (PLHIV). Proper implementation of TB infection control (TBIC) practices in health care facilities can curb TB menace among PLHIV and the public. We assessed the implementation of TB infection control in health care facilities offering Anti-Retroviral Therapy (ART) in Enugu State, Nigeria.

**Methods:**

we employed a cross-sectional research design and assessed TB infection control practices in nine State owned public health care facilities offering antiretroviral therapy (ART) services for PLHIV. A 23 item World Health Organization (WHO) checklist for infection control in health care facilities was used to collect data. We assessed the five minimum standards as well as the four sets of TB infection control (TBIC) measures. Frequencies, percentages and chi square statistic were used to analyze data.

**Results:**

only four (44%) health care facilities that provides ART services studied in Enugu State implemented TBIC practices. Higher proportion of the rural and secondary facilities implemented TBIC although the difference is not statistically significant (p>0.05). Implementation was better with the administrative controls while the personal protective equipment was almost non-existent.

**Conclusion:**

less than half of the facilities offering ART services in the Enugu State have TB infection control measures. We therefore recommend that in order to reduce TB infection among PLHIV, the issue of proper TBIC in health care facilities need urgent attention. Materials provision, staff training and retraining are issues that must be tackled to achieve the aim of reduction of TB infection among PLHIV, health care workers and the public.

## Introduction

Tuberculosis (TB) remains the commonest opportunistic infection among people living with HIV (PLHIV). People living with HIV are more likely to develop TB than people without HIV due to immune suppression associated with HIV infection [1]. In 2019, 27,000 people in Nigeria died of HIV-associated TB and there were approximately 46,000 new cases of TB/HIV co-infection in the country [2]. A 30-month prevalence of TB among HIV clients at Global Fund supported facilities in Nigeria is 7.7 per cent [3]. In Enugu State, infections especially from HIV and AIDS and TB alone or as co infection and septicemia were the most common causes of death in medical ward in a tertiary hospital in the State [4]. Therefore, one of the activities to reduce the burden of TB among PLHIV in the WHO policy on collaborative TB/HIV activities is to implement TB infection control measures in health care facilities, congregate settings and households.

Tuberculosis infection control (TBIC) is a combination of methods designed to minimize the risk of TB transmission within population [5]. The WHO guideline on TBIC in health care facilities has four sets of measures for facility level TBIC. These include: managerial activities; administrative control; environmental control; and personal protective equipment [6]. Among these measures are five key standards that reflect the demonstrable minimum measures that are consistent with international guidelines [7]. All of the key standards must be in existence for the facility to qualify as having TBIC practices. Proper implementation of these measures in ART facilities will help to reduce drastically the incidence of TB among PLHIV, health care workers and the general public.

Implementation is concerned with carrying out the programme activities as originally intended. Factors like unavailability of personnel and equipment, knowledge and motivation level of the personnel may affect carrying out the programme activities as originally intended [8]. Health care workers in Enugu State who are supposed to implement TBIC in health care facilities lack motivation and incentive to carry out their duties effectively [9,10]. These might affect the implementation of TBIC in health care facilities in Enugu State. Studies on infection control measures have been carried out in Nigeria [11-13]. However, none of the studies was carried out in ART clinics and none applied the five WHO minimum key standards for TBIC. We utilized the five key standards as well as the four sets of measures [7]. The purpose of this study was therefore to assess the implementation of TBIC measures in health care facilities offering ART services in Enugu State. Specifically, we determined: 1) Proportion of health facilities offering ART services that implement TB infection control measures in Enugu State; 2) proportion of health facilities offering ART services that implement TB infection control measures in Enugu State based on facility location and facility type.

**Hypotheses:** there is no significant difference in the proportion of health facilities offering ART services that implement TB infection control measures in Enugu State according to facility location and facility type.

## Methods

**Study design and setting:** we employed cross sectional research design and conducted the study in Enugu State, Nigeria. Enugu State is the capital of the old eastern region of Nigeria. Health care system in the State is organized according to the national health care policy (primary, secondary and tertiary). However, comprehensive care and support services including ART services for PLHIV are provided only at the general and teaching hospitals. These hospitals represent the secondary and tertiary levels of care. Tuberculosis and HIV services are totally free in public health care facilities. We carried out this study in Enugu State owned public health care facilities because most of the PLHIV access care in these facilities. Some private and mission health care facilities also provide ART services but sometimes, cost is attached to their services, so limiting the number of people accessing care in their facilities. There are nine health care facilities (eight general hospitals, and one teaching hospital) in Enugu State providing the comprehensive care and support including ART to PLHIV. We studied the nine facilities. Some HIV services like HIV testing and counseling, prevention of mother to child transmission (PMTCT) of HIV are provided at the primary level health care but these facilities do not provide the comprehensive care and support like the secondary and tertiary levels of care. There are also several private and mission health care facilities situated throughout the State that also provides health care services including HIV care for PLHIV [14]. The study population comprised of all the nine State owned public health care facilities offering ART services.

Eligibility criteria includes public health facility that is offering anti-retroviral therapy for people living with HIV in Enugu State. No sampling was done because the population is small and manageable therefore all the nine facilities were studied. The variables of the study were health care facility location and facility type. Location was characterized into urban and rural while type of facilities studied were secondary and tertiary facilities.

**Data collection:** in order to find out implementation of each standard of TBIC, we interviewed TB focal persons in the health care facilities using an adapted WHO checklist for periodic evaluation of TBIC in health care facilities to collect data [7]. In addition to the interview, we also used observations, facility registers and other means of verification stipulated in the checklist to verify the responses of the interviewees. The checklist consisted of 23 item WHO TBIC standards grouped into four parts: managerial with six items; administrative with ten items; environmental with five items; and personal protective equipment with two items. The checklist has a response option of “yes” or “no” with means of verification of the responses (Annex 1). A health facility was considered to have implemented TBIC if it met all the five recommended minimum standard for a facility to qualify as having infection control practice consistent with international guidelines. These standards were written in bold fonts in the checklist and include: there is written facility specific infection control plan (that includes TB infection control); there is designated person responsible for implementing TBIC practices in the facility; TB symptoms occurring among staff are immediately investigated and if TB is diagnosed, is treated, registered and reported in the confidential occupational health records or TB register; patients with cough are identified on arrival at the facility, given guidance in cough etiquette, separated from other patients and fast tracked through all waiting areas including consultation, investigation and drug collection; and waiting area is well ventilated (windows and doors open when feasible) and there is clear display of messages on cough hygiene in all areas frequented by patients. Data were collected between February and May, 2019.

**Statistical analysis:** the Statistical Package for Social Sciences (SPSS) version 22.0 was used for all the statistical analyses. Frequency counts and percentages were used to analyse the categorical variables and their significant difference were examined using Chi-square test. The probability values less than 0.05 (p<0.05) were considered significant.

**Ethical considerations:** ethical approval for this study was granted by the research ethics committee of Enugu State Ministry of Health (MH/MSD/REC19/050). The research was conducted in accordance with the World Medical Association Declaration of Helsinki. Health facilities were given codes to ensure anonymity.

## Results

A total of nine health care facilities were assessed in this study. Of the nine facilities, three were located in urban areas while six were in rural locations. Also, eight of the facilities were secondary level care facilities while one was a tertiary facility. Table 1 show that only four facilities (44%) implemented all the five minimum standards and therefore met the WHO minimum standard for a facility to qualify as having infection control practice consistent with international guidelines. This means that only 44 per cent of health facilities offering ART services studied in Enugu State implemented TB infection control practices. Furthermore, the table shows that patients with cough identified, triaged and fast tracked is practiced in 9 (100%) of the facilities. Waiting area well ventilated and message on cough hygiene displayed is practiced by 7 (78%) of the facilities. Also, 6 (67%) of the facilities have designated person responsible for implementing TBIC in the facility and TB symptoms among staff are immediately investigated, treated and reported. Only 5 (56%) of facilities have facility specific TB infection control plan.

**Table 1:** proportion of health care facilities offering ART services that implement WHO minimum standard for TB infection control in Enugu State

S/N	Health care facility code	Has TB infection control plan	Person in charge of TBIC	TB infection among staff immediately investigated and reported	Patients with cough identified, triaged and fast tracked	Waiting area well ventilated and message on cough hygiene displayed	Achieved minimum standard
1	AWGH	1	1	1	1	1	1
2	UDGH	1	1	1	1	1	1
3	EEGH	1	1	1	1	1	1
4	NSGH	1	1	1	1	1	1
5	PGHA	0	0	0	1	1	0
6	AGGH	0	0	1	1	1	0
7	IKGH	0	1	0	1	0	0
8	ORGH	0	0	0	1	1	0
9	ETHP	1	1	1	1	0	0
Total %		55.5	66.6	66.6	100	77.7	44.4

Key: 1: yes, 0: no

AWGH: Awgu General Hospital; UDGH: Udi General Hospital; EEGH: Enugu Ezike General Hospital; NSGH: Nsukka General Hospital; PGHA: Poly General Hospital Asata; AGGH: Agbani General Hospital; IKGH: Ikem General Hospital; ORGH: Oji River General Hospital; ETHP: Enugu State University of Science and Technology Teaching Hospital Parklane

Table 2 shows that higher proportion of rural facilities 3 (50%) than the urban facilities 1 (33%) implemented all the five minimum standards therefore met the WHO minimum standard for TB infection control consistent with international guidelines. The table also show that 4 (50%) of the secondary facility implemented WHO minimum standard for TB infection control consistent with international standards while the tertiary facility studied did not implement TB infection control measures. However, no significant difference existed in infection control practice between rural and urban health care facilities as well as between secondary and tertiary health care facilities (p>0.05).

**Table 2:** summary of Chi-square analysis of difference in proportion of health care facilities that implement WHO minimum standard of TB infection control based on facility location and facility type

Variable	Total number of health care facility	Number achieving minimum standard	Percentage	Chi square (χ^2^)	Degree of freedom	p- value
**Location of facility**						
Urban	3	1	33.3			
Rural	6	3	50	0.228	1	0.633
**Type of facility**						
Secondary	8	4	50			
Tertiary	1	0	0	1.275	1	0.259
**Total**	9	4	44.4			

* significant at p<0.05

Table 3 further illustrates the proportion of health facilities in the State that practiced all the standards stipulated in the WHO checklist for periodic evaluation of TBIC in health care facilities. The table show that for the managerial control: 6 (67%) of the facilities have TB symptoms occurring among staff immediately investigated and if TB is diagnosed, is treated, registered and reported in the confidential occupational health records or in TB register; 5 (56%) of facilities have facility specific infection control plan and designated person responsible for implementing TBIC practice in the facility. However, only 3 (33%) facilities had designated TBIC focal person received documented TBIC training or refresher training within the last two years while only 2 (22%) facilities had all clinic staff received documented TBIC training or refresher training within the last two years.

**Table 3:** number of health care facilities that implement all the WHO standards for TB infection control in Enugu State

S/N	Standards	Frequency	Percentage
	**Managerial**		
1	There is written facility-specific infection control plan (that includes TB infection control (TBIC))	5	55.6
2	There is a budget allocated for TB infection control activities	4	44.4
3	There is a designated person (and committee in larger facilities) responsible for implementing TBIC practices in the facility	5	55.6
4	Designated TBIC focal person has received documented TBIC training or refresher training within the last two years	3	33.3
5	all clinic staff have received documented TBIC training or refresher training within the last two years	2	22.2
6	TB symptoms occurring among staff are immediately investigated and if TB is diagnosed, is treated, registered and reported in the confidential occupational health records or in TB register	6	66.7
	**Administrative**		
7	Patients with cough are identified on arrival at the facility, given guidance on cough etiquette, separated from other patients and fast tracked through all waiting areas including consultation, investigations and drug collection	9	100
8	All information and education material is systematically checked to prevent stigmatizing or discriminatory language	9	100
9	TB information for patients is readily available and offered by staff	9	100
10	Supplies are readily available for coughing patients (tissues, surgical masks) and are being used, and there are medical waste bins for safe disposal	6	66.7
11	A package of HIV and HIV associated TB prevention and care is available for staff on site; (1) confidential HIV testing and post exposure prophylaxis for all staff; and (2) antiretroviral therapy ART and isoniazid preventive therapy IPT for HIV positive staff	5	55.6
12	There is a tracking mechanism (e.g. registers) and person responsible for monitoring turnaround time from TB screening to diagnosis and from TB diagnosis to treatment initiation	7	77.8
13	The median time between screening positive for TB symptoms and actual diagnosis is no more than one day	5	55.6
14	The median time between actual diagnosis and treatment initiation is no more than one day	8	88.9
15	WHO recommended rapid diagnostics is the first TB diagnostics test for PLHIV	5	55.6
16	HIV testing is offered to all patients with presumptive TB and evaluation for time to start ART is carried out if found HIV positive	9	100
	**Environmental**		
17	The facility design, patient flow and triage system comply with what is outlined in the infection control policy	7	77.8
18	Waiting area is well ventilated (i.e. windows and doors open when feasible) and there is clear display of messages on cough hygiene in all areas frequented by patients	7	77.8
19	Patients are not crowded in hallways or waiting areas	7	77.8
20	Sputum samples are collected in a well-ventilated, clearly designated area away from others preferably outdoors	8	88.9
21	Diagnosed TB cases, who are hospitalized are isolated or grouped according to drug sensitivity status in rooms with adequate natural ventilation	5	55.6
	**Personal protective equipment**		
22	Respirators are readily available for and being used by staff, particularly for high-risk aerosol generating procedures and for providing care to patients with diagnosed or suspected infections as per national guide lines	1	11.1
23	Staff have been trained in the proper fit and use of respirators	1	11.1

For the administrative controls, four out of the ten standards: HIV testing is offered to all patients with cough; TB information for patients is readily available; all information and education material is checked; and patients with cough are identified on arrival were implemented by all the 9 (100%) facilities studied. For the environmental controls, 8 (89%) facilities had sputum samples collected in a well-ventilated, clearly designated area away from others preferably outdoors while three of the standards; the facility design, patient flow and triage system comply with what is outlined in the infection control policy; waiting area is well ventilated and there is clear display of messages on cough hygiene in all areas frequented by patients; and patients are not crowded in hallways or waiting areas were practiced by 7 (78%) of the facilities. For the personal protective equipment, only 1 (11%) facility had respirators readily available and staff trained on proper fit and use of respirators.

## Discussion

This study revealed that only four facilities (44%) met the WHO minimum standard for a facility to qualify as having infection control practice consistent with international standard (Table 1). This means that less than half of health facilities offering ART services in Enugu State implemented TB infection control practices. The WHO has a set of five infection control standards that a facility must meet to qualify as implementing infection control measures consistent with international standard and less than half of the health facilities in Enugu State met the criteria. The implication of this finding can be disastrous for TB control in the State. It means that more than half of the facilities offering ART services in Enugu State do not have infection control practices. If TB is not controlled in ART facilities where PLHIV access care, the rate of TB infection among PLHIV might increase thereby jeopardizing the efforts of TB control agencies. The objective (to reduce TB among PLHIV) might be far from being achieved. However, the finding is consistent with some other studies. For instance, none of the health facilities in Enugu State had all the infection control measures in place [12]; the proportion of health facilities that have proper TB infection control practice was low at 38 per cent in Northwest Ethiopia [15]. Although the above studies were carried out in primary health facilities, the present study which was carried out in secondary and tertiary health facilities where PLHIV access care needed to have a more robust TBIC practices. The findings of this study do not augur well for TB control in the study area and globally.

Higher proportion of rural facilities (3, 50%) than the urban facilities (1, 33%) implemented the WHO minimum standards for TB infection control. Also 4 (50%) of the secondary health care facilities implement WHO minimum standards for TB infection control while the tertiary facility studied did not meet the criteria to qualify as having infection control measures because only four out of the five key standards were in existence in the facility (Table 2). Although no significant difference was seen in the implementation of TB infection control between rural and urban facilities as well as between secondary and tertiary facilities (p >0.05), urban and tertiary health care facilities in the State need to be worked on in terms of TB infection control practices. It seems more attention is focused on rural and secondary health care facilities. For a tertiary institution in the State not to qualify as having TB infection control practice is a thing of concern. The management of this tertiary health care facility should as a matter of urgency address the issue of infection control in the facility so as to meet the WHO minimum standard for a facility to qualify as having infection control practice.

The results in Table 3 also revealed that on implementation of facility level managerial measures, only 5 (56%) facilities have facility specific infection control plan, 6 (67%) facilities have designated person responsible for implementing TBIC in the facility, and TB symptoms among staff are immediately investigated, treated and reported. This finding is at variance and better than some other findings in Nigeria. For instance, only 1 (8.3%) facility had documented TB infection control policy and 5 (41.7%) had TBIC officer in facilities offering joint TB/HIV services in German Leprosy and Tuberculosis Relief Association (GLRA) supported States in Nigeria [11]. None of the clinics in Ikeja, Lagos, Nigeria had specific infection control plan [13]. However, this finding is similar to finding in South Africa where it is reported that 43.3 per cent of clinics did not have specific infection control plan which means that 56.7 percent have infection control plan just like the finding of our study [16] while in another study in the same country, 26 (63.4%) had infection control committee [17]. Our study however, showed that training of health care workers on TBIC was poor as only 3 (33%) facilities had designated TBIC focal person received documented TBIC training or refresher training within the last two years while only 2 (22%) facilities had all clinic staff received documented TBIC training or refresher training within the last two years. The findings of our study suggest an improvement in the implementation of some of the facility level managerial measures except staff training in the area, which need to be sustained and improved upon to ensure proper TBIC in health care facilities. The issue of staff training should not be neglected if reduction of TB among PLHIV is to be achieved.

Four out of the ten administrative measures assessed in the WHO checklist were practiced by all the facilities-patients with cough are identified on arrival, given cough etiquette, separated from others and fast tracked through all waiting area 9 (100%) together with other three controls. This finding is encouraging and needs to be sustained. The finding is at variance and better than most other studies. For instance, only one facility (8.3%) had health workers checking for coughing GLRA supported facilities in Nigeria [11]; no facility was consistently screening patients for cough in Ikeja, Nigeria [13]; 48.8 per cent of facilities did not separate coughing patients from other patients in South Africa [16] while only 11 (26.8%) separated coughing and non-coughing patients in another study in South Africa [17]. The environmental control practice was good as 8 (89%) facilities had sputum samples collected in a well-ventilated, clearly designated area away from others preferably outdoors while three of the standards; the facility design, patient flow and triage system comply with what is outlined in the infection control policy; waiting area is well ventilated and there is clear display of messages on cough hygiene in all areas frequented by patients; and patients are not crowded in hallways or waiting areas were practiced by 7 (78%) of the facilities. This can however, be improved on to support the administrative controls in minimizing the risk of TB infection among PLHIV. This proportion is higher than other studies. For instance, no facility had Information Education Communication (IEC) material in Nigeria [11], only 18.9 per cent had open windows in South Africa (16), 60 per cent of facilities had adequate ventilation in Ikeja, Lagos, Nigeria, 73.2 per cent had open windows in another study in South Africa [17].

Furthermore, Table 3 showed that the personal protective measures were existent in only one facility (11%) and only one facility had its staff received any training on personal protective equipment. Personal protective equipment is meant to protect health care workers from contracting TB as they carry out their duties. Their nonexistent will likely expose health care workers to tuberculosis infection thereby affecting manpower in the facilities. This finding is similar with other findings in Africa. Only 4 (20%) facilities in Ikeja, Nigeria had N 95 respirator [13]; only one-third of nurses and one in ten of community health workers in South Africa had received training on proper fit and use of respirators [16]. Personal protective equipment were not practiced well in Northwest Ethiopia [15].

This study has shown the status of different sets of TB infection control in health care facilities offering ART in the study area using the WHO standardized checklist with means of verification of the different aspects of TBIC. This has added to existing literature on the subject matter and the results could be used by relevant stakeholders to improve TBIC in health care facilities. However, the study failed to explore other reasons likely to contribute to lapses in TBIC in health care facilities and this becomes a limitation of the study. Future research therefore needs to explore health care workers´ as well as health care system characteristics that may affect TBIC implementation in health care facilities offering ART considering the public health importance of TB/HIV co-infection.

## Conclusion

Less than half of health care facilities offering ART services in Enugu State implemented TBIC measures. Rural and secondary health care facilities practiced infection control more than the urban and tertiary health care facilities although the difference is not statistically significant. The administrative controls were better practiced while the personal protective equipment was almost nonexistent except in one facility. Training and refresher training of TB focal person and all clinic staff was poor in the health care facilities studied. Therefore, we recommend that TBIC in health care facilities offering ART services in Enugu State need serious improvement by addressing the issues of personal protective equipment and staff training on TBIC. The administrative control practices need to be sustained but there is still room for improvement in some of the administrative controls. These can be achieved by regular provisions of necessary materials and training of staff on TBIC by both government and non-governmental agencies in order to curb the menace of TB infection among PLHIV and general community members.

### What is known about this topic

- Tuberculosis infection control in health care facilities is one of the activities recommended by WHO to reduce the burden of TB among PLHIV;
- People living with HIV are more susceptible to TB infection because their immunity are compromised;
- Proper implementation of TB infection control measures will reduce TB among PLHIV and the public.

### What this study adds

- Implementation of TB infection control in health care facilities offering ART in Enugu State is poor especially with the personal protective equipment;
- Health care workers in ART clinics in Enugu State do not have sufficient training and retraining on TB infection control practices.

## Annex

**Annex 1**: data collection tool: checklist for periodic evaluation of tb infection control in health care facilities (to be collected by the researcher/research assistants) (PDF-102Kb)
